# Supplementary material for: Mechanism and structural dynamics of sulfur transfer during de novo [2Fe-2S] cluster assembly on ISCU2
Source: Nat Commun. 2024 Apr 16;15:3269. doi: 10.1038/s41467-024-47310-8 (PMC11021402; doi:10.1038/s41467-024-47310-8)
Supplement: Supplementary file 1 — Supplementary Information [file 41467_2024_47310_MOESM1_ESM.pdf]

Supplementary Information

for

Mechanism and structural dynamics of sulfur transfer during *de novo* [2Fe-2S] cluster assembly on ISCU2

Vinzent Schulz, Ralf Steinhilper, Jonathan Oltmanns, Sven-A. Freibert,
Nils Krapoth, Uwe Linne, Sonja Welsch, Maren H. Hoock,
Volker Schünemann, Bonnie J. Murphy, Roland Lill

This file contains:
13 Supplementary Figures
5 Supplementary Tables
Supplementary References

Supplementary Figures

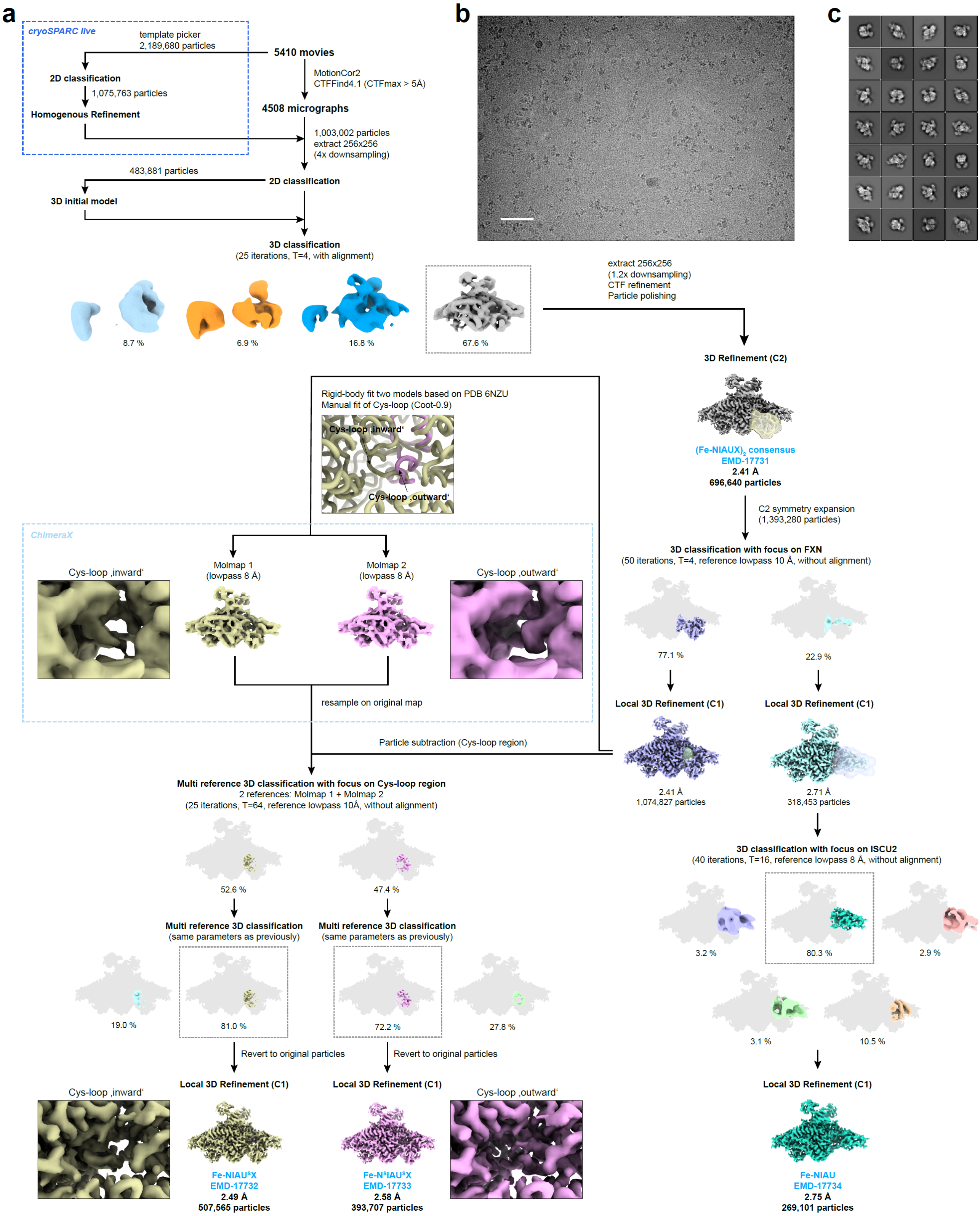

Supplementary Fig. 1: Cryo-EM image processing. a, Visualisation of the major processing steps as described in the Methods section. Unless stated otherwise, processing was performed in RELION-3.1. **b**, Representative cryo-EM micrograph (scale bar 500 Å). **c**, Representative 2D class averages.

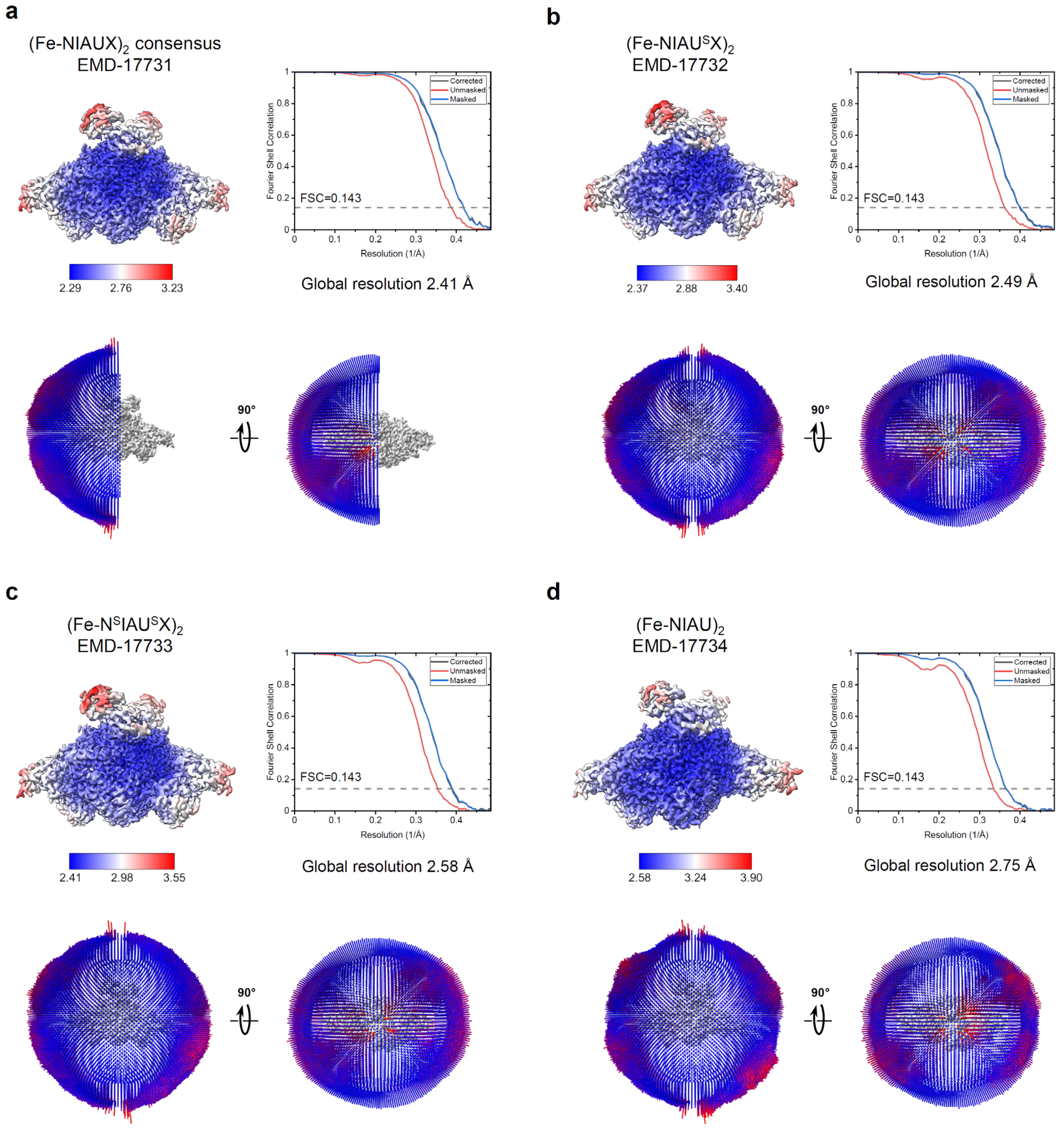

Supplementary Fig. 2: 3D reconstructions. Local resolution estimation, Fourier Shell Correlation (FSC) curves and angular distribution plots for **a**, $(Fe\text{-}NIAUX)_2$ consensus (EMD-17731), **b**, $(Fe\text{-}NIAU^{S}X)_2$ (EMD-17732), **c**, $(Fe\text{-}N^{S}IAU^{S}X)_2$ (EMD-17733), and **d**, $(Fe\text{-}NIAU)_2$ (EMD-17734).

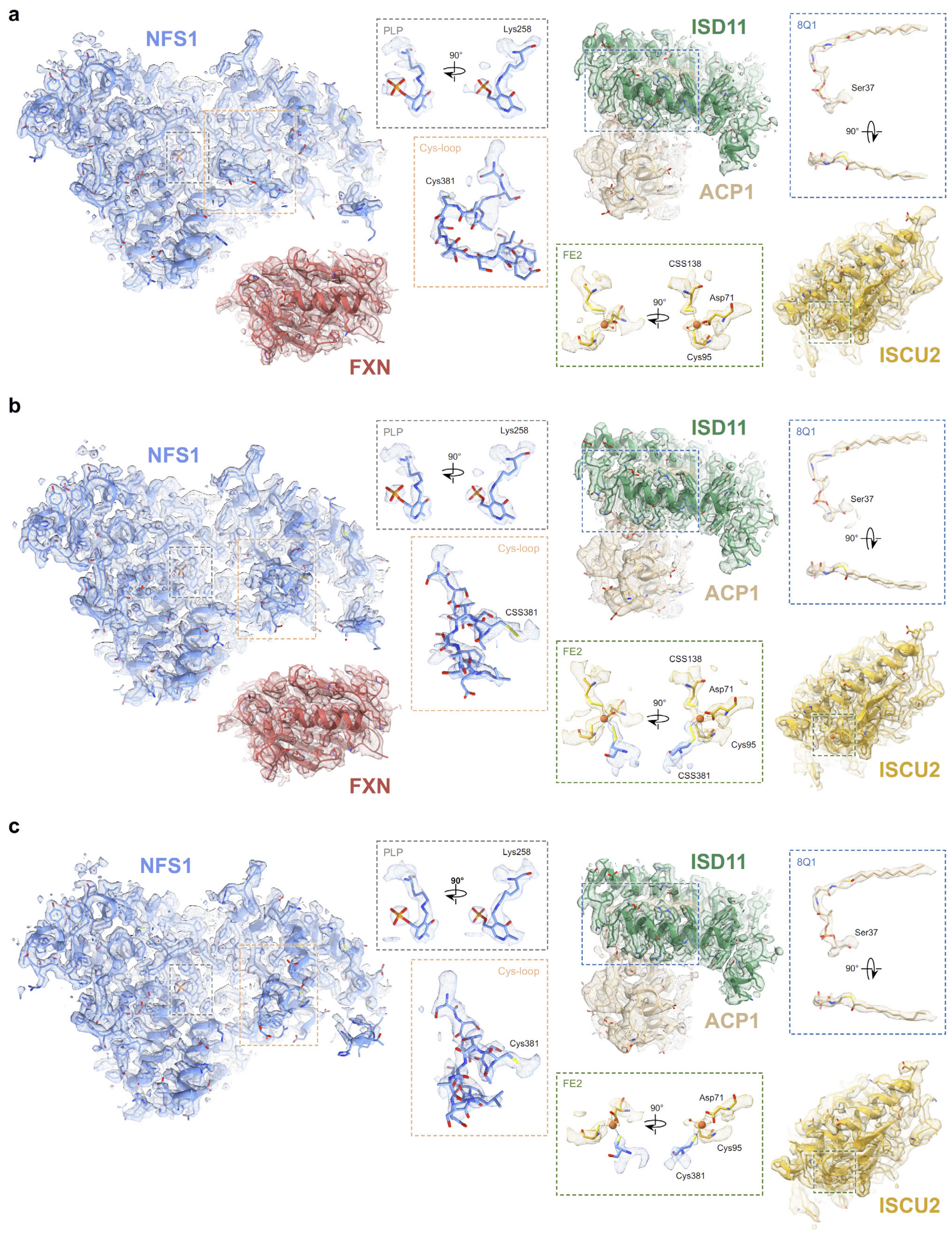

Supplementary Fig. 3: Atomic models and maps of all subunits and cofactors. a, (Fe-NIAU^{S}X)$_2$ (PDB 8PK8; EMD-17732). **b**, (Fe-N^{S}IAU^{S}X)$_2$ (PDB 8PK9; EMD-17733). **c**, (Fe-NIAU)$_2$ (PDB 8PKA; EMD-17734).

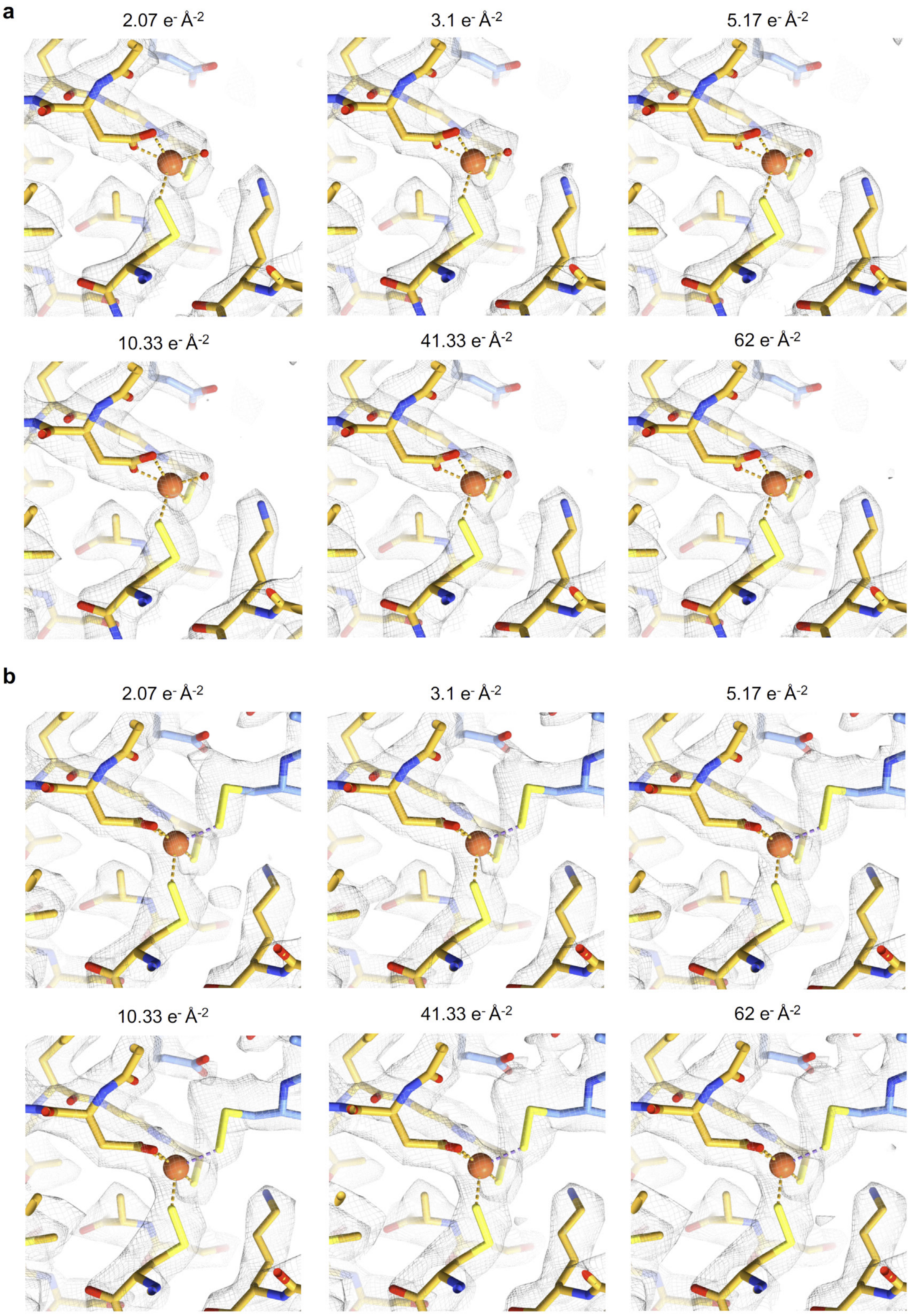

Supplementary Fig. 4: Cryo-EM maps of the ISCU2 cluster assembly site at increasing accumulated electron dose. 3D reconstructions from truncated cryo-EM movies and atomic model of the ISCU2 cluster assembly site of **a**, (Fe-NIAU^{S}X)$_2$ with persulfidated Cys138^{ISCU2}, and **b**, (Fe-N^{S}IAU^{S}X)$_2$ with persulfidated Cys381^{NFS1} and Cys138^{ISCU2}. Maps are displayed as a grey mesh at 4 σ, models are displayed as sticks. Low-dose reconstructions are fully consistent with the atomic models built using dose-weighted, full-frame reconstructions.

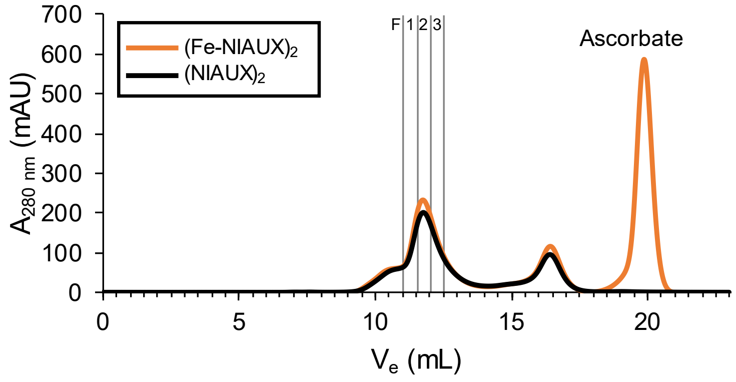

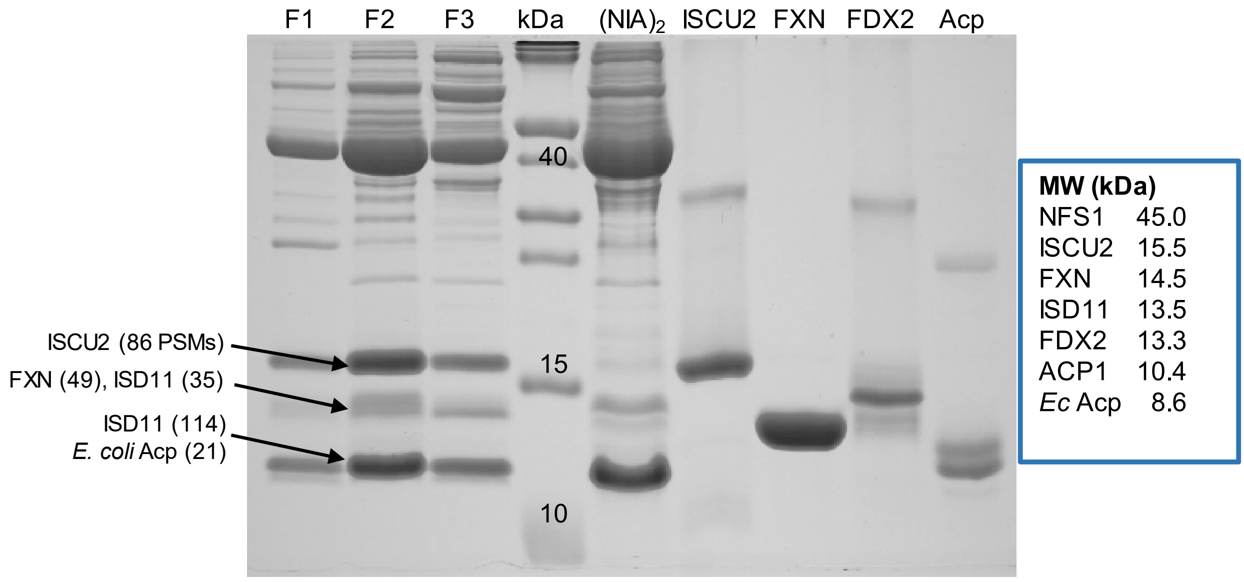

Supplementary Fig. 5: ISCU2 binds more stably to the core ISC complex than FXN. a, $(NIA)_2$ (50 µM), ISCU2, and FXN (100 µM each) were incubated with either 500 µM $FeCl_2$ and 500 µM ascorbate (red, $(Fe\text{-}NIAUX)_2$) or 1 mM DTPA Fe chelator (black, $(NIAUX)_2$) and incubated at room temperature for 10 min. Samples were subjected to analytical SEC and fractions (F1 – F3) of the indicated $(NIAUX)_2$ peak were subjected to tricine SDS-PAGE shown in (**b**). Individual purified proteins were also run to facilitate protein identification. Bands marked by arrows were further analyzed by qualitative MS. Peptide spectrum matches (PSMs) are given in brackets for the detected proteins. ISD11 produced two distinct bands upon SDS-PAGE separation. Human ACP1 generally shows low coverage in qualitative MS analysis and only *E. coli* Acp peptides were detected. Bands for ISD11 and FXN ran at similar positions in the gel. The experiment reveals stable binding of ISCU2 to $(NIA)_2$. In contrast, lower amounts of bound FXN could be detected, indicating less stable binding to $(NIAU)_2$.

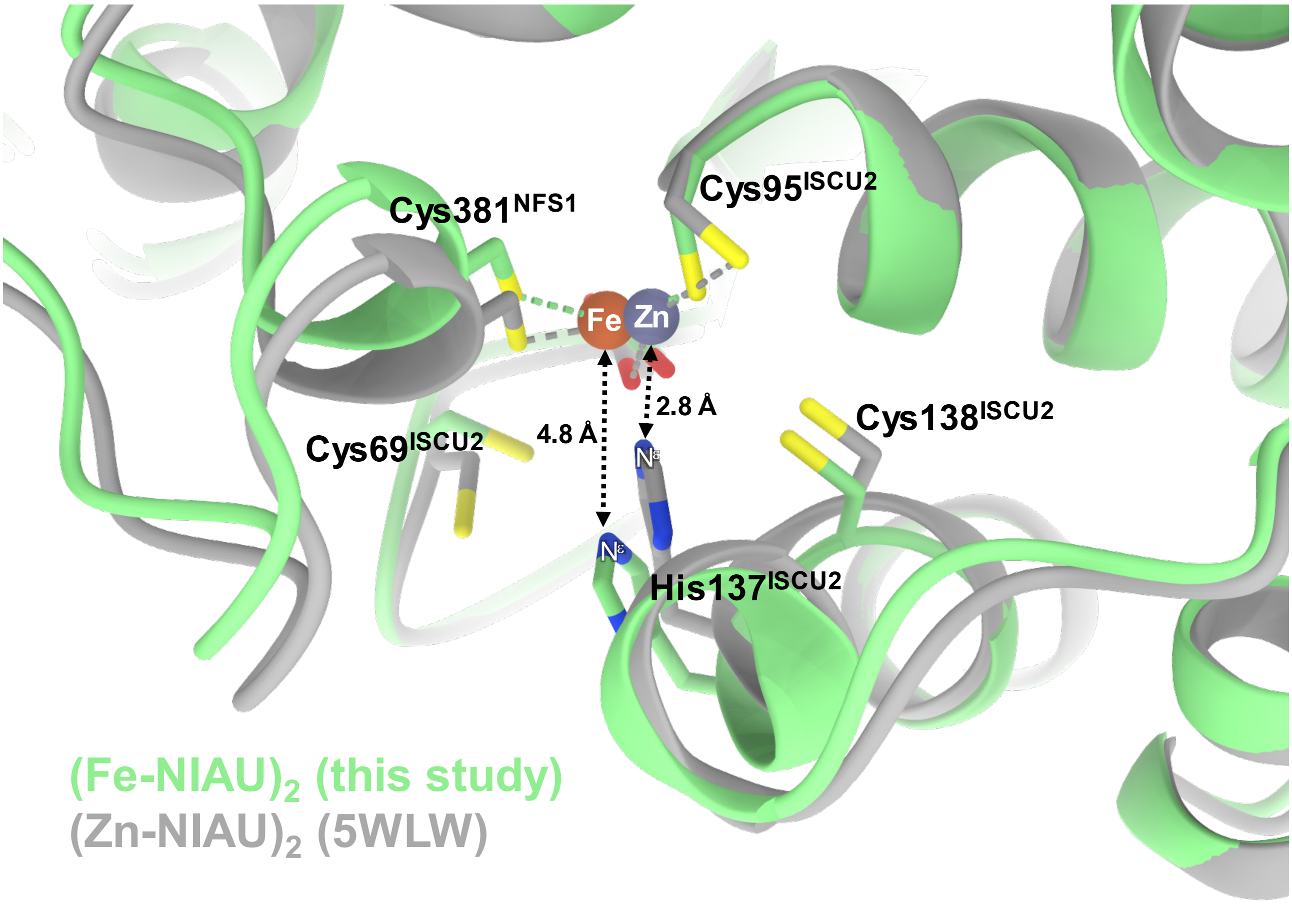

Supplementary Fig. 6: Structural rearrangements at the ISCU2 cluster assembly site. Comparison of the ISCU2 cluster assembly site within the structures of $(Fe\text{-}NIAU)_2$ (this study) and $(Zn\text{-}NIAU)_2$ [1]. In these structures, Cys138^{ISCU2} is positioned far from the sulfur-donating Cys381^{NFS1}. His137^{ISCU2} is positioned between Cys381^{NFS1} and Cys138^{ISCU2} thereby hindering direct sulfur transfer.

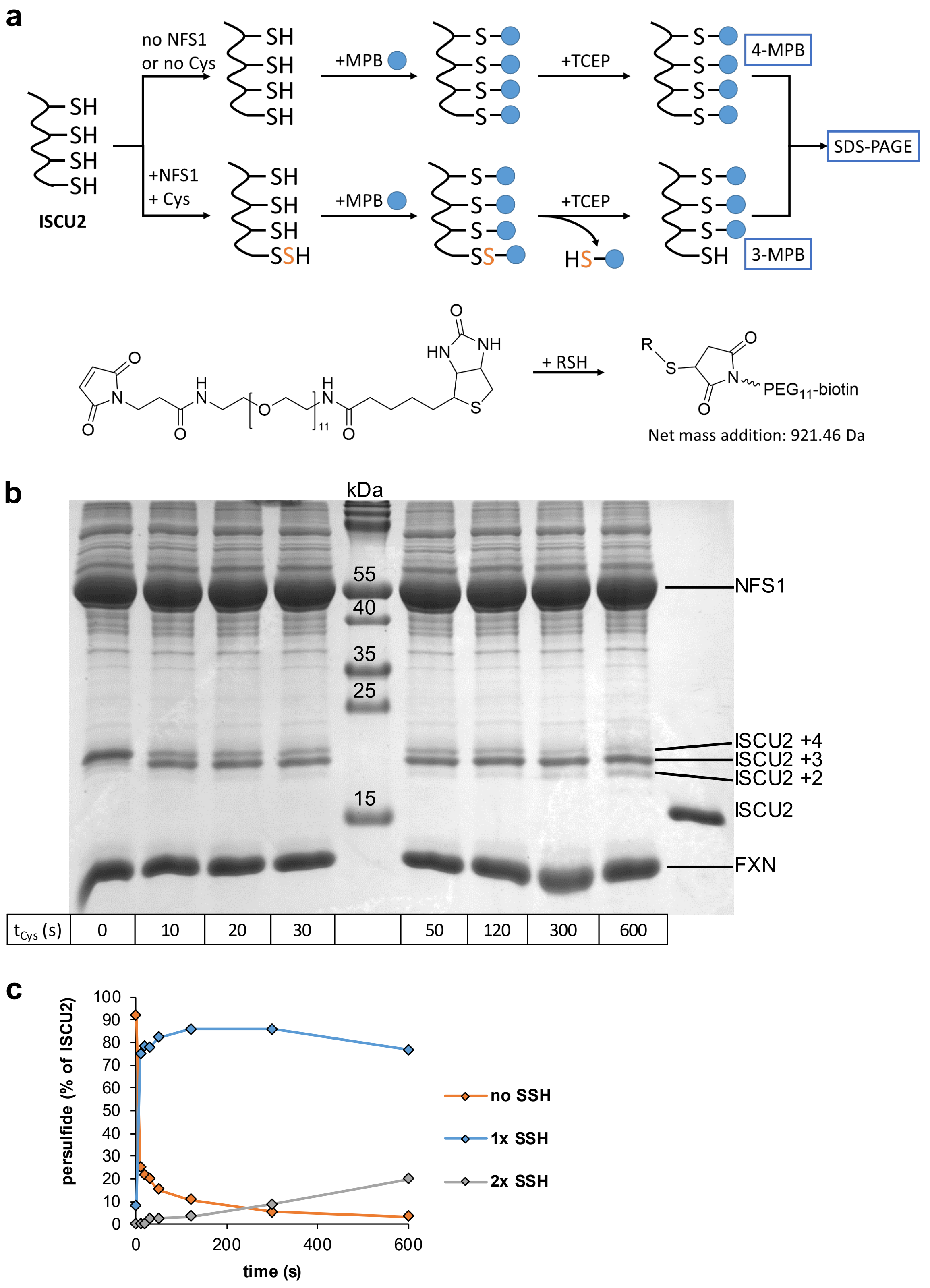

Supplementary Fig. 7: Time course of enzymatic ISCU2 persulfidation *in vitro* reveals physiological and non-physiological persulfidation products. a, Schematic outline of the MPB-based ISCU2 persulfidation assay. ISCU2 containing four Cys residues (-SH) is incubated with excess cysteine desulfurase complex $(NIA)_2$, FXN, Fe^{2+} (to assure that all ISCU2 is bound in the $(Fe\text{-}NIAUX)_2$ complex) and ascorbate (to maintain reduced Fe^{2+}). Addition of free Cys rapidly induces

persulfide formation on Cys381^{NFS1} under concomitant release of alanine (not shown). Cys381^{NFS1} then transfers the persulfide sulfur to a Cys residue on ISCU2. The reaction is quenched by adding maleimide-polyethylenglycol$_{11}$-biotin (MPB), which labels all thiols including persulfides and leads to a net mass addition of 921 Da per molecule MPB. Addition of SDS denatures all proteins, ensuring that the biochemical reaction is quickly stopped and that otherwise sterically hindered Cys residues are labelled completely. Subsequently, all persulfides are cleaved using SDS-PAGE sample buffer containing the reductant TCEP, leading to a loss of the persulfide-bound MPB moiety. Control reactions lacking either $(NIA)_2$ or Cys do not generate persulfides, and yield fully MPB-labelled ISCU2. The mass shift induced by the release of MPB from ISCU2 can be visualized by SDS-PAGE, and indicates the number of persulfidated Cys residues (one in the example). **b**, In reactions containing 20 µM ISCU2, 20 µM $(NIA)_2$, 40 µM FXN, as well as 100 µM Fe^{2+} and ascorbate, persulfide formation was initiated by addition of excess Cys. After the indicated time periods reactions were quenched by addition of MPB and SDS. Persulfides were cleaved by adding TCEP, and the persulfidation-induced mass shift of ISCU2 was analyzed by SDS-PAGE. The outmost right lane shows non-labelled ISCU2 and the outmost left lane (reaction without Cys) ISCU2 with all four protein Cys residues labelled with MPB (ISCU2 +4). ISCU2 +3 and +2 correspond to species with one and two persulfidated protein Cys residues, respectively. **c**, ISCU2 species with no, one or two persulfides (SSH) within the gel depicted in (**b**) were quantified by densitometry to show the time course of persulfidation.

a

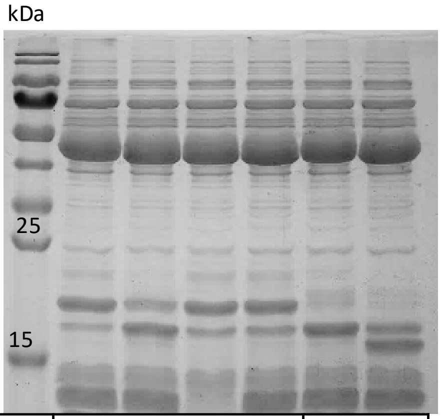

ISCU2	WT				C130S	
Fe(II)	+	+	+	-	+	+
FXN	+	+	-	+	+	+
Cys	-	+	+	+	-	+

b

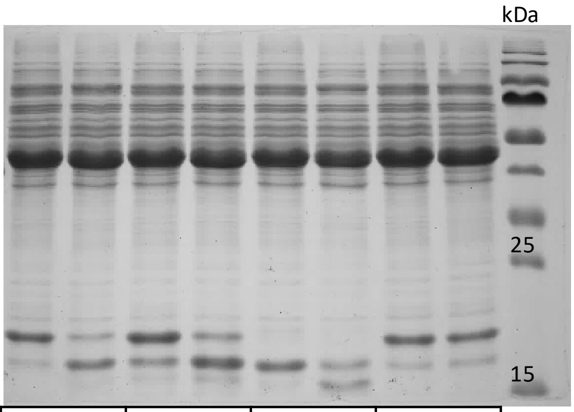

ISCU2	WT		n.a.		C69S		D71A	
Cys	-	+			-	+	-	+

c

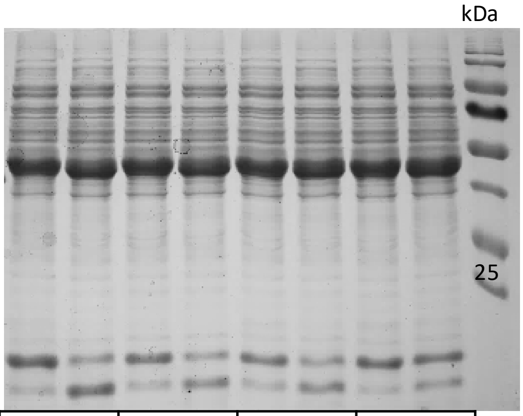

ISCU2	n.a.		H137A		n.a.		n.a.	
Cys			-	+				

d

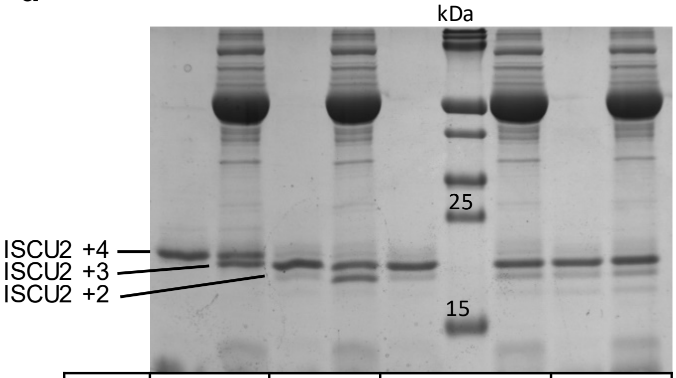

ISCU2	WT		C69S		C95S			C138S	
NIA	-	+	-	+	-		+	-	+

e

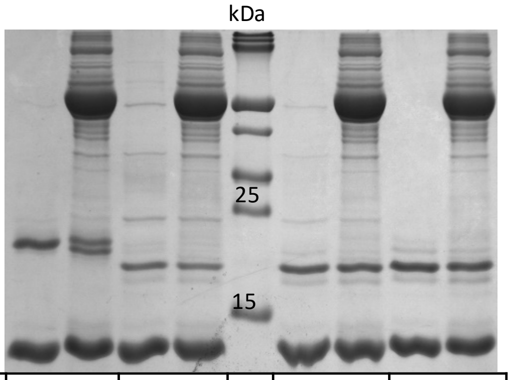

ISCU2	WT		C95/138S			C69/138S		C69/95S	
NIA	-	+	-	+		-	+	-	+

Supplementary Fig. 8: MPB-based persulfidation analysis of ISCU2 and variants. a-e, Standard reactions contained wild-type (WT) or various variant ISCU2 proteins, as well as FXN, $(NIA)_2$, ascorbate and Fe^{2+}. Reactions were initiated by Cys addition, and were quenched with MPB and SDS after 10 s. Several negative controls were performed by omitting either $(NIA)_2$ or Cys. In addition, reactions containing WT ISCU2 and lacking either FXN or Fe^{2+}/ascorbate were performed

as indicated in (**a**), the latter containing 1 mM DTPA for Fe chelation. The non-persulfidated species becomes labelled with four molecules MPB (ISCU2 +4) in WT ISCU2 containing four Cys residues. Upon exchange of one or two Cys residues, ISCU2 +3 and ISCU2 +2, respectively, represent the non-persulfidated species. Molecular mass markers are given in kDa. Abbreviation: n.a., not applied.

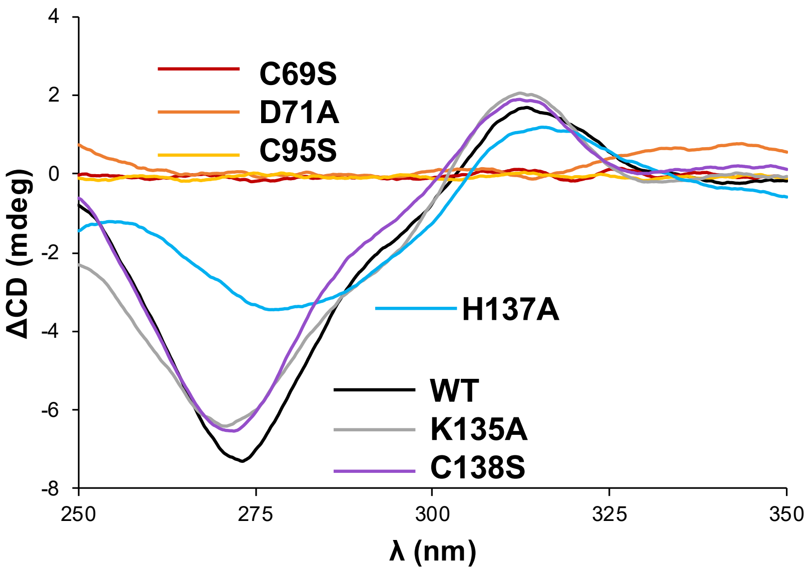

Supplementary Fig. 9: CD-spectroscopic analysis of ISCU2 cluster assembly-site mutant proteins reveals residues required for Fe ligation. CD spectra of ISCU2 (WT) and mutant proteins were recorded before and after addition of 2 eq. Fe^{2+} and corresponding difference spectra are shown. All samples contained 100 µM ISCU2 and 2 mM TCEP and were measured 2 min after mixing the components.

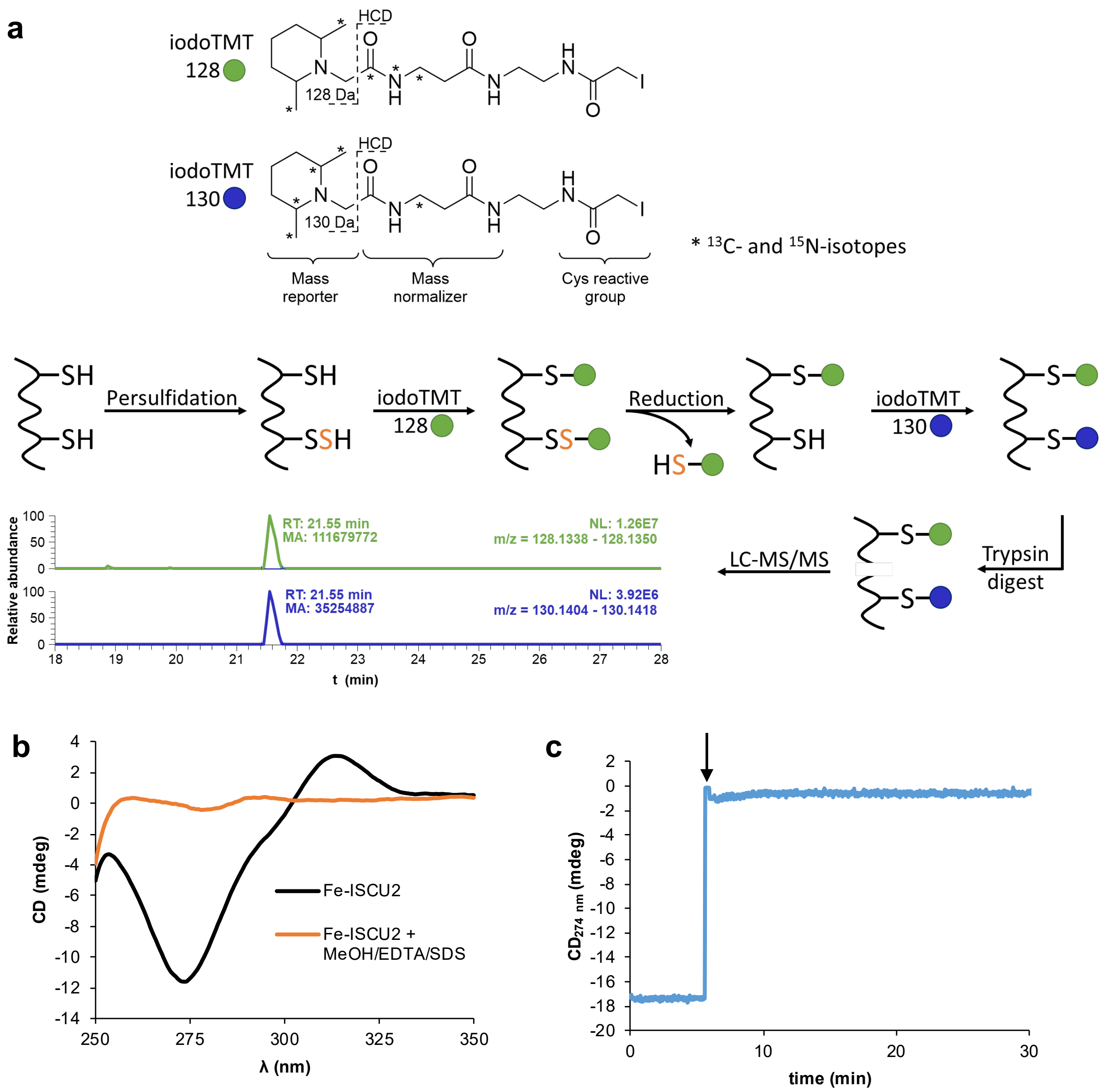

Supplementary Fig. 10: Analysis of persulfidation of individual Cys residues via an iodoTMT-based assay. a, Upper panel: The iodoTMT sixplex kit (Thermo Fisher) includes six labels of identical structure but with differing distribution of ^{13}C- and ^{15}N-isotopes, named iodoTMT 126, 127, 128, 129, 130 and 131, according to the mass of the reporter moiety. Labels form a covalent C-S bond with thiols via the Cys-reactive group. Depicted are iodoTMT 128 and 130 as examples. Lower panel: Schematic outline of the iodoTMT-based persulfidation assay. **Step 1:** ISCU2 Cys residues were persulfidated by the cysteine desulfurase NFS1 in the presence of free Cys. **Step 2:** Persulfidated samples were labelled with, e.g., iodoTMT 128. Label stocks containing SDS and EDTA facilitated simultaneous labelling and rapid denaturation of the proteins to avoid a prolonged persulfidation reaction. Excess label was quenched by adding excess Cys. **Step 3:** Persulfides were reductively cleaved *in situ* using TCEP. **Step 4:** Cleaved samples were subjected to a second

labelling step (e.g., iodoTMT 130), and excess label was quenched with Cys. **Step 5:** Labelled proteins were cleaved by on-bead trypsin digest and resulting peptides enriched according to the manufacturer's protocol. **Step 6:** NanoHPLC-MS^2 enables separation of peptides and fragmentation of iodoTMT molecules by higher energy collisional dissociation or collision-induced dissociation (HCD or CID, indicated by dashed lines). IodoTMT molecules were fragmented by HCD, and signals of mass reporter fragments were integrated to calculate the percentage of persulfidation for each Cys-containing peptide. **b,c**, A quench mixture used in iodoTMT-based persulfidation assays leads to rapid removal of Fe from Fe-ISCU2. 100 µM ISCU2, 200 µM $FeCl_2$ and 2 mM TCEP were mixed under anaerobic conditions to a final volume of 300 µL in a cuvette which was tightly sealed with a rubber plug. The CD spectrum of Fe-ISCU2 was recorded (**b**, black curve). Subsequently, the CD signal at 274 nm was recorded in a time course (**c**). After 5.5 min (marked by an arrow in **c**), the CD spectrometer was opened and 300 µL of a quench mixture (50% v/v methanol, 10 mM EDTA and 10% w/v SDS) as used in iodoTMT-based persulfidation assays was injected into the cuvette as quickly as possible. The CD spectrometer was closed again 10 s after the injection (the shortest possible time), at which time point the Fe-ISCU2 CD signal had almost completely vanished. After completion of the time course, a CD spectrum was recorded, confirming loss of Fe from ISCU2 (**b**, orange curve). The experiment indicates that application of the quench mix as done in the iodoTMT-based analysis of Fe-dependent ISCU2 persulfidation leads to the rapid release of Fe from Fe-ISCU2, abolishing persulfidation. High concentrations of methanol and SDS likely induce fast protein denaturation. As the 10 s time point represents the technical limitation of this experiment, loss of Fe may proceed even faster.

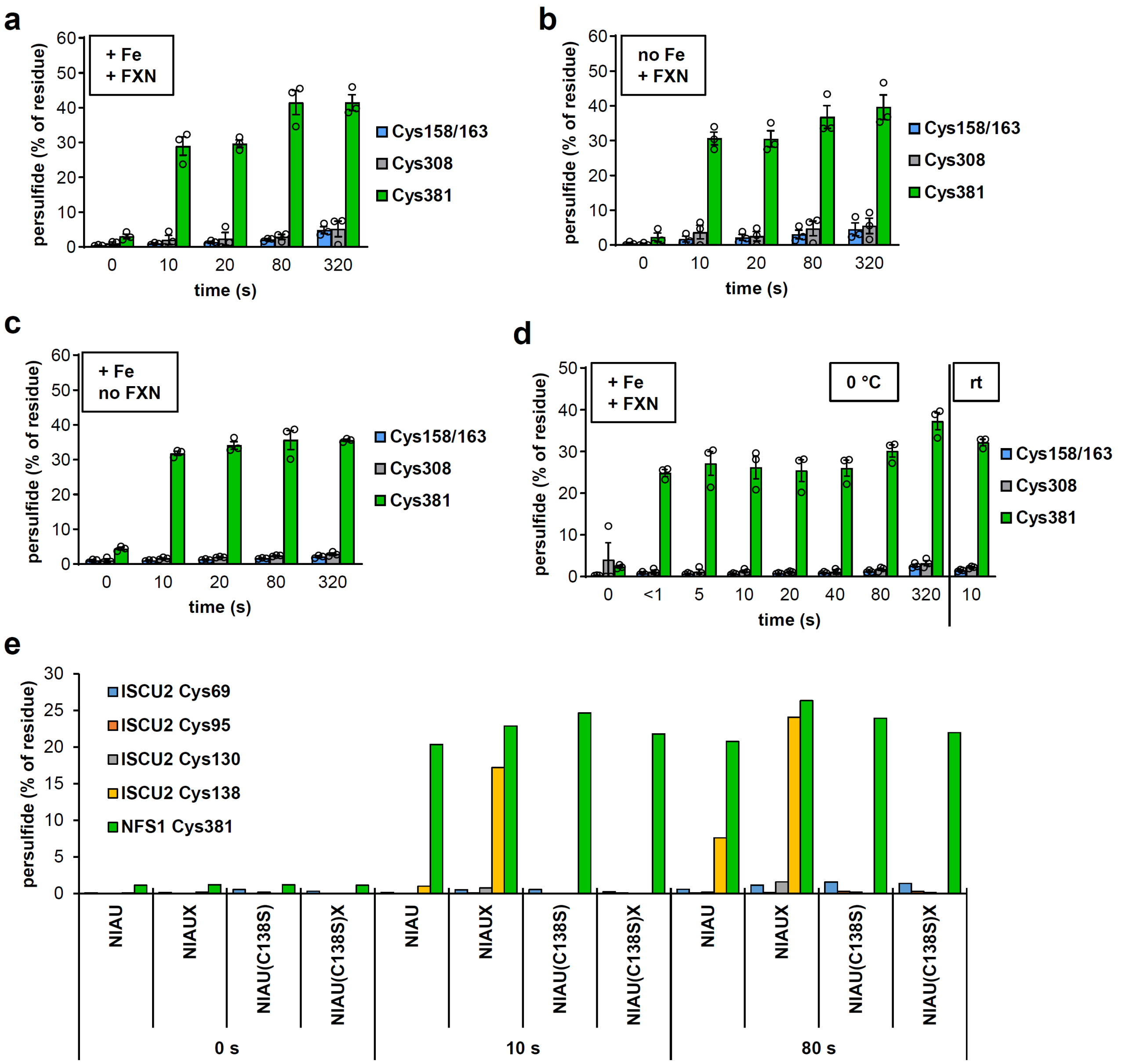

Supplementary Fig. 11: Analysis of persulfidation of Cys residues of NFS1 and ISCU2 via iodoTMT. a-d, Persulfidation of the indicated NFS1 Cys residues was quantified from the same reactions shown in Fig. 3b-e according to the procedure described in Supplementary Fig. 10. Error bars indicate the SEM (n = 3; independent experiments). rt, room temperature. **e**, Persulfidation of indicated Cys residues of ISCU2 or ISCU2-C138S and of NFS1 employing different core ISC complexes at the indicated reaction times performed at room temperature. Generally, reactions contained 30 µM ISCU2 WT or ISCU2-C138S (in e), 15 µM FXN and 7.5 µM $(NIA)_2$ as indicated, as well as 150 µM $FeCl_2$ and 75 µM cysteine. IodoTMT-based labelling and HPLC-MS^2 analysis were performed as described above. Source data are provided as a Source Data file. U: ISCU2, X: FXN.

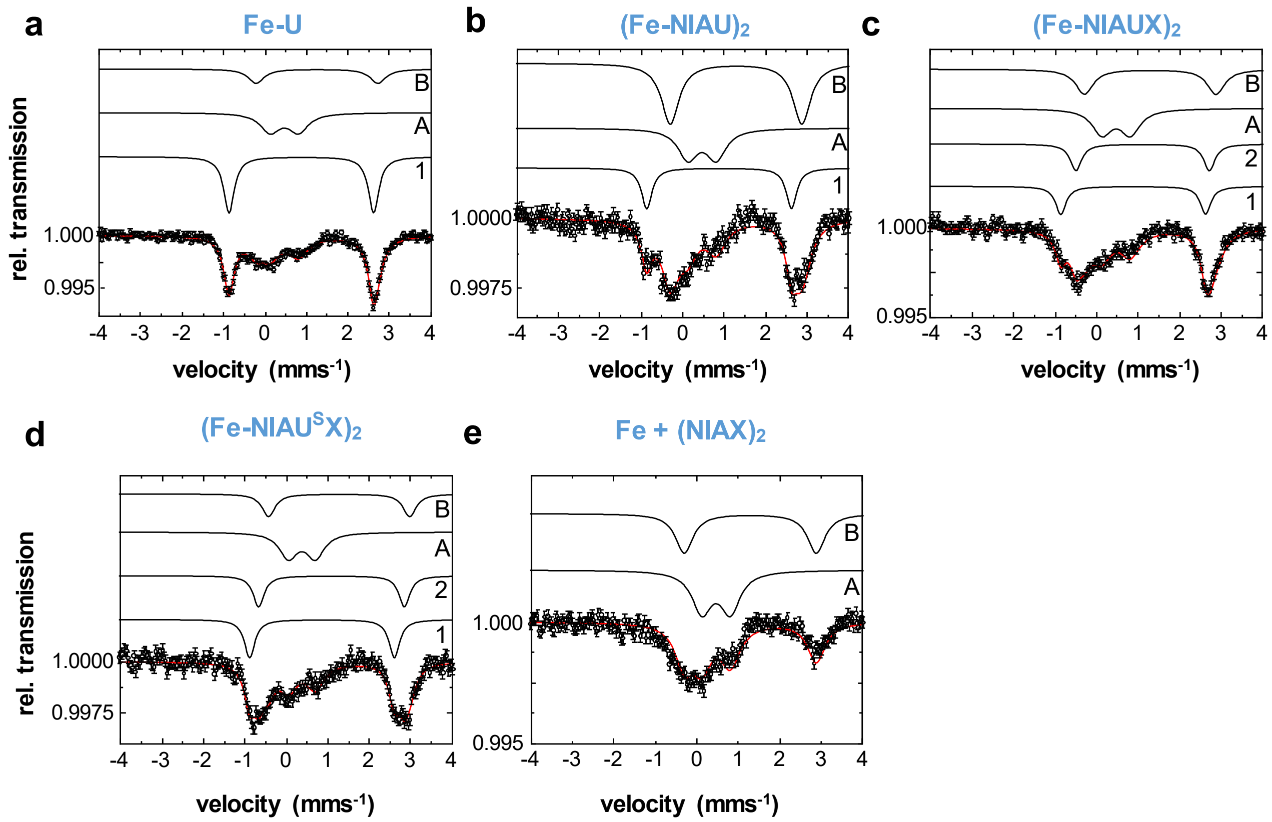

Supplementary Fig. 12: Mössbauer spectroscopic analysis of intermediates of [2Fe-2S] cluster synthesis before optimization of complex formation conditions. Mössbauer spectra of the indicated proteins or complexes reconstituted with 1.5 eq. ^{57}Fe-enriched ferric ammonium citrate over ISCU2, i.e. under non-optimized buffer conditions as shown in Fig. 4, Table 3. Cysteine was added as the final component in (**d**) to initiate ISCU2 persulfidation. U: ISCU2; X: FXN; F: FDX2. Proteins were present in a ratio NIA:U:X of 1:1:2. Each spectrum was recorded at 77 K and zero applied external field. The red lines display the best fit of the data using the components shown as black lines with parameters given in Supplementary Table 1. Mössbauer data points are presented as relative transmission per velocity channel derived from detector counts (see Methods). Error bars indicate the +/- SD.

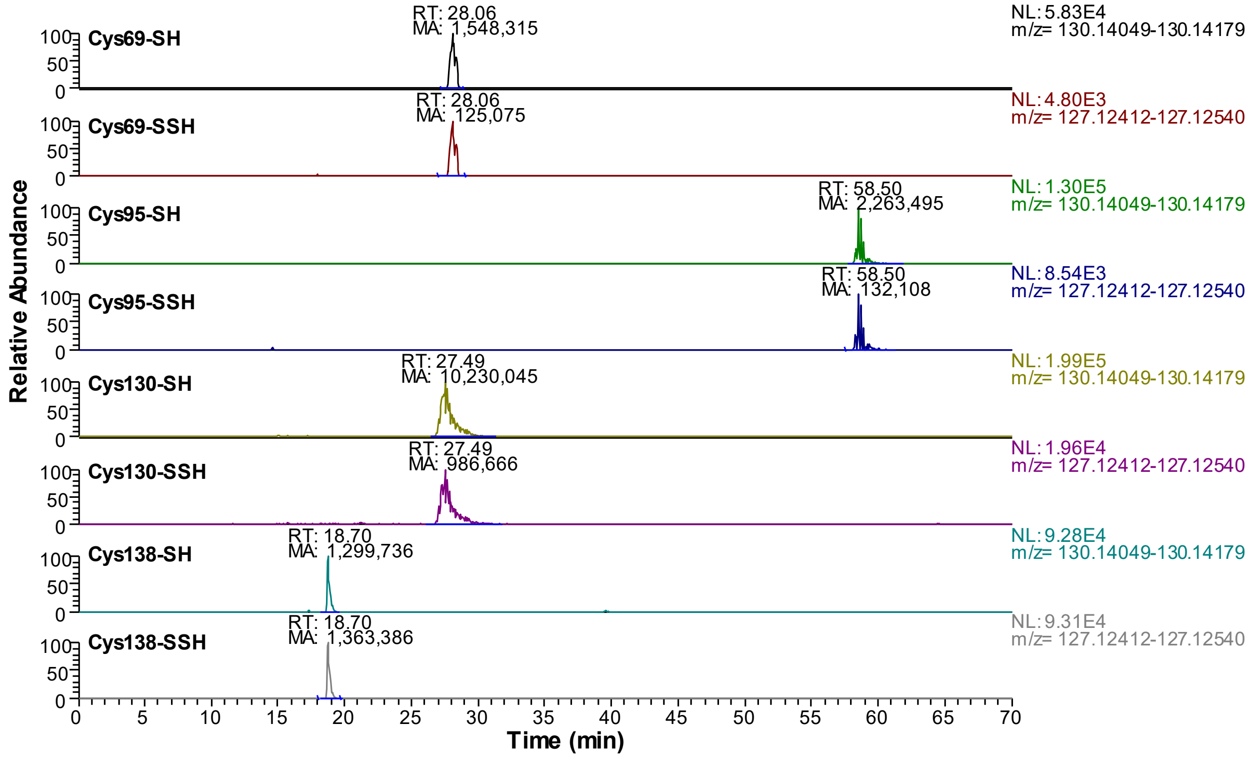

Supplementary Figure 13: MS^2 ion traces of Cys-containing ISCU2 peptides. Representative data referring to Fig. 3b (t = 320 s) show the analyzed persulfidated (SSH) and non-persulfidated (SH) Cys species. The retention time (RT) and mass area (MA) of the peaks integrated for quantification of persulfidation according to Supplementary Fig. 10a are indicated. The normalization level (NL) and the m/z range for the targeted iodoTMT MS^2 fragments are shown on the right.

Supplementary Tables

Supplementary Table 1: Mössbauer parameters of ^{57}Fe- bound ISCU2 or various ISC complex intermediates.

	Component 1				Component 2				Component A				Component B			
	δ	ΔE_Q	Γ	A^2 (%)	δ	ΔE_Q	Γ	A^2 (%)	δ	ΔE_Q	Γ	A^2 (%)	δ	ΔE_Q	Γ	A^2 (%)
**Fe-U**	0.87	3.49	0.31	49					0.46	0.68	0.57	31	1.25	2.94	0.46	20
**$(Fe\text{-}NIAU)_2$**	0.87	3.49	0.31	21					0.46	0.68	0.57	28	1.28	3.18	0.51	51
**$(Fe\text{-}NIAUX)_2$**	0.87	3.49	0.31	19	1.10	3.22	0.33	21	0.46	0.68	0.57	32	1.28	3.18	0.51	28
**$(Fe\text{-}NIAU^{S}X)_2$**	0.87	3.49	0.31	25	1.15	3.53	0.34	30	0.46	0.68	0.57	28	1.32	2.94	0.51	17
**$Fe + (NIAX)_2$**									0.46	0.68	0.57	53	1.28	3.18	0.51	47

Isomer shift (δ), quadrupole splitting (ΔE_Q) and line width (Γ) are given in mm s^{-1}. A^2, relative spectral area given in % for the experiment shown in Supplementary Fig. 12. ISCU2-specific components 1 and 2 exhibit parameters similar to those of the respective $(NH_4)_2{}^{57}Fe(SO_4)_2$-reconstituted samples (Table 3). A difference is evident for $(Fe\text{-}NIAU)_2$, exhibiting 27% component 2 in $(NH_4)_2{}^{57}Fe(SO_4)_2$-reconstituted $(Fe\text{-}NIAU)_2$ (Table 3), but component 2 is absent here in . Components A and B exhibit a high line width, indicating unspecific Fe binding. Their presence in the $Fe+(NIAX)_2$ sample shows these species to be independent of ISCU2.

Supplementary Table 2: Composition of samples used for Mössbauer spectroscopy.

a

Compound (µM)	Fe-U	(Fe-NIAU)$_2$	(Fe-NIAUX)$_2$	(Fe-NIAU^{S}X)$_2$	Fe + (NIAX)$_2$	[2Fe-2S]-U
**ISCU2**	900	300	300	300	-	450
**Na-ascorbate**	2700	900	900	900	900	2700
**$(NH_4)_2{}^{57}Fe(SO_4)_2$**	900	300	300	300	300	900
**NIA**	-	450	450	450	450	22.5
**FXN**	-	-	450	450	450	22.5
**FDX2**	-	-	-	-	-	22.5
**FDXR**	-	-	-	-	-	0.23
**NADPH**	-	-	-	-	-	2250
**$MgCl_2$**	-	-	-	-	-	2250
**cysteine**	-	-	-	900	-	900

b

compound	Fe-U	(Fe-NIAU)$_2$	(Fe-NIAUX)$_2$	(Fe-NIAU^{S}X)$_2$	Fe + (NIAX)$_2$
**TCEP**	2000	2000	2000	-	2000
**ISCU2**	350	350	350	350	-
**Na-ascorbate**	5250	5250	5250	5250	5250
**^{57}Fe (FAC)**	525	525	525	525	525
**NIA**	-	350	350	350	350
**FXN**	-	-	700	700	700
**cysteine**	-	-	-	350	-

Sample composition from Mössbauer experiments shown in **a**) Fig. 4, Table 3 and **b**) Supplementary Fig. 12, Supplementary Table 1 are compiled.

Supplementary Table 3: Plasmid constructs.

Plasmid	ORF	Backbone	Reference
pASK-IBA43(+)-*FDX2*	*FDX2 (1-68Δ)*	pASK-IBA43(+)	2
pET24b(+)-*ISCU2*	*ISCU2-His$_6$ (1-34Δ)*	pET24b(+)	3
pETDuet1-*NFS1-ISD11*	*NFS1 (1-55Δ), His$_6$-Tev-ISD11*	pETDuet1	3
pRSFDuet1-*ACP*	*ACP (1-68Δ)*	pRSFDuet1	3
pMCSG7-*FXN*	*His$_6$-Tev-FXN (1-80Δ)*	pMCSG7	3
pETDuet1-*FDXR*	*His$_6$-FDXR (1-32Δ)*	pETDuet1	4

Plasmid constructs used in this study are based on the canonical sequences extracted from the Uniprot database (uniprot.org).

Supplementary Table 4: Buffer compositions used for protein purification.

His buffer	His buffer ISCU2 D71A	AEC buffer	AEC elution buffer	SEC buffer	SEC buffer $(NIA)_2$
50 mM Tris	50 mM HEPES	35 mM Tris	35 mM Tris	50 mM Tris	50 mM Tris
300 mM NaCl	500 mM KCl	50 mM NaCl	1 M NaCl	150 mM NaCl	150 mM NaCl
5% (w/v) glycerol	10% (w/v) glycerol	5% (w/v) glycerol	5% (w/v) glycerol	5% (w/v) glycerol	10% (w/v) glycerol
pH 7.4 with HCl	5 mM TCEP	pH 7.5 with HCl	pH 8.0 with HCl	pH 7.4 with HCl	pH 8.0 with HCl
	pH 7.5 with KOH				

Supplementary Table 5: Final concentrations of additives during protein purifications.

ISCU2, FXN	ISCU2 D71A	$(NIA)_2$
10 mM TCEP	10 mM TCEP	10 mM TCEP
10 mM DTPA	10 mM DTPA	10 mM DTPA
25 mM KCN	25 mM KCN	0.5 mM PLP
	10 mM DT	
	40 mM $MgCl_2$	
	10 mM ATP	

Supplementary References

1. Boniecki MT, Freibert SA, Muhlenhoff U, Lill R, Cygler M. Structure and functional dynamics of the mitochondrial Fe/S cluster synthesis complex. *Nat Commun* **8**, 1287 (2017).

2. Schulz V*, et al.* Functional spectrum and specificity of mitochondrial ferredoxins FDX1 and FDX2. *Nat Chem Biol* **19**, 206-217 (2023).

3. Freibert SA*, et al.* N-terminal tyrosine of ISCU2 triggers [2Fe-2S] cluster synthesis by ISCU2 dimerization. *Nat Commun* **12**, 6902 (2021).

4. Sheftel AD*, et al.* Humans possess two mitochondrial ferredoxins, Fdx1 and Fdx2, with distinct roles in steroidogenesis, heme, and Fe/S cluster biosynthesis. *Proc Natl Acad Sci U S A* **107**, 11775-11780 (2010).
