# Supplementary material for: Mechanism and structural dynamics of sulfur transfer during de novo [2Fe-2S] cluster assembly on ISCU2
Source: Nat Commun. 2024 Apr 16;15:3269. doi: 10.1038/s41467-024-47310-8 (PMC11021402; doi:10.1038/s41467-024-47310-8)
Supplement: Supplementary file 3 — Description of Additional Supplementary Files [file 41467_2024_47310_MOESM3_ESM.pdf]

File name: Supplementary Movie 1

Description: The conformational changes induced by binding of FXN to the $(FeNIAU)_2$ complex. Morph between the Fe-NIAU (PDB 8PKA) and the Fe-NIAUX (PDB 8PK8) structures. Selected residues in NFS1, ISCU2 and FXN are displayed.
